# Supplementary material for: Clinical and Virological Characteristics and Prognostic Factors in Viral Necrotizing Retinitis
Source: J Pers Med. 2022 Oct 29;12(11):1785. doi: 10.3390/jpm12111785 (PMC9695359; doi:10.3390/jpm12111785)
Supplement: Supplementary file 1 [file jpm-12-01785-s001.zip › jpm-1906748-supplementary.pdf]

Supplemental Table S1. Baseline and evolving characteristics according to disease presentation					
**Variables (described in terms of patients)**	**ARN (N=20)**	**PORN (N=4)**	**CMV retinitis (N=13)**	**Total (N=37)**	**P-value**
**Females, N (%)**	9/20 (45%)	2/4 (50%)	3/13 (23.1%)	14/37 (37.8%)	0.48
**Age (years), Mean (SD)**	55.4 (20.1)	53.5 (11.7)	59.6 (17.9)	56.7 (18.4)	0.73
**Immunodepression, N (%)**	0/19 (0%)	4/4 (100%)	13/13 (100%)	17/36 (47.2%)	**0.0005**
**Bilateralization of viral necrotizing retinitis, N (%)**	4/20 (20%)	1/4 (25%)	5/13 (38.5%)	10/37 (27%)	0.59
**Time to bilateralization from 1st visit (months), Mean (SD)**	66.3 (44.9)	0	11 (20.9)	32 (41.8)	0.07
**Variables (described in terms of eyes)**	**ARN (N= 21)**	**PORN (N=5)**	**CMV retinitis (N=15)**	**Total (N=41)**	
**VZV, N (%)**	32% (13/41)	12% (5/41)	0% (0/41)	44% (18/41)	**<0.001**
**HSV2, N (%)**	19% (8/41)	0% (0/41)	0% (0/41)	19% (8/41)	
**CMV, N (%)**	0% (0/41)	0% (0/41)	37% (15/41)	37% (15/41)	
**Baseline BCVA (LogMAR), Mean (SD)**	1.0 (0.8)	0.9 (0.7)	0.9 (0.8)	0.9 (0.8)	0.58
**Vitritis grade at baseline, Mean (SD)**	2.4 (0.9)	2 (0)	1.6 (1.5)	2.1 (1.1)	0.11
**Number of quadrants affected by viral necrotizing retinitis at baseline, Mean (SD)**	2.1 (1.4)	2.6 (1.5)	1.9 (1.2)	2.1 (1.3)	0.72
**Time from symptom onset to initiation of treatment (days), Mean (SD)**	9.1 (13.7)	7 (4.9)	54.2 (73.5)	21.7 (44.4)	**0.0018**
**Duration of IV treatment (days), Mean (SD)**	19.7 (8.3)	22 (12.5)	29 (29.2)	23.5 (18.2)	0.87
**IV Aciclovir, N (%)**	13/21 (61.9%)	5/5 (100%)	4/12 (33.3%)	22/38 (57.9%)	**0.04**
**IV Ganciclovir, N (%)**	0/21 (0%)	0/5 (0%)	8/13 (61.5%)	8/39 (20.5%)	**0.0005**
**IV Foscarnet, N (%)**	15/21 (71.4%)	3/5 (60%)	6/13 (46.2%)	24/39 (61.5%)	0.32
**Number of Ganciclovir IVIs, Mean (SD)**	4.8 (4.9)	8.2 (4.3)	9.2 (10.9)	6.8 (7.7)	0.19
**Number of Foscarnet IVIs, Mean (SD)**	5.1 (7.9)	1.6 (3.6)	1.7 (4.5)	3.4 (6.5)	0.23
**Retinal detachment during follow-up, N (%)**	8/21 (38.1%)	0/5 (0%)	3/15 (20%)	11/41 (26.8%)	0.22

**Time from baseline to retinal detachment (days), Mean (SD)**	91.2 (110.0)	-	119 (183.2)	98.8 (123.9)	0.92

HSV:herpes simplex virus; VZV: varicella zoster virus; CMV: cytomegalovirus; ARN: acute retinal necrosis ; PORN: progressive outer retinal necrosis; F: female; N : number; % : percentage ; SD : standard deviation; IQR: interquartile range(1st- 3rd interquartile). BCVA: best corrected visual acuity; IVI: intravitreal injection; IV: intravenous. P-value is for ARN versus PORN versus CMV retinitis.

Supplemental Table S2. Characteristics of patients and eyes according to immune status and causative virus

	**Immunodepression**			**Immunocompetence**			**p-value [a]**	**p-value [b]**
	**CMV**	**VZV**	**Total**	**HSV2**	**VZV**	**Total**		
**Variables (described in terms of patients)**	**(N=13)**	**(N=4)**	**(N=17)**	**(N=7)**	**(N=12)**	**(N=19)**		
**Gender (females), N(%)**	3 / 13 (23.1%)	2 / 4 (50%)	5 / 17 (29.4%)	3 / 7 (42.9%)	6 / 12 (50%)	9 / 19 (47.4%)	0.27	0.56
**Age (years), Mean (SD)**	59.6 (17.9)	53.5 (11.7)	58.2 (16.5)	39.1 (13.6)	67.2 (14.7)	56.8 (19.6)	0.89	***0.014***
**Follow-up (months), Mean (SD)**	10.8 (10.9)	5.2 (5.9)	9.5 (10.1)	24.6 (12.0)	12.2 (14.1)	16.8 (14.4)	0.18	0.14
**Bilateral disease, N(%)**	5 / 13 (38.5%)	1 / 4 (25%)	6 / 17 (35.3%)	2 / 7 (28.6%)	1 / 12 (8.3%)	3 / 19 (15.8%)	0.25	0.35
**Time to bilateralization (months), Mean (SD)**	11 (20.9)	0 (NA)	9.2 (19.2)	84 (16.9)	1 (NA)	56.3 (49.4)	***0.059***	0.17
**Variables (described in terms of eyes)**	**(N=15)**	**(N=5)**	**(N=20)**	**(N=8)**	**(N=13)**	**(N=21)**	**p-value [a]**	**p-value [b]**
**Baseline BCVA (LogMAR), Mean (SD)**	0.90 (0.84)	0.92 (0.74)	0.90 (0.80)	0.79 (0.71)	1.20 (0.85)	1.04 (0.81)	0.31	0.44
**BCVA at Month 1 (LogMAR), Mean (SD)**	1.11 (0.96)	0.70 (0.76)	0.99 (0.90)	0.64 (0.57)	1.78 (0.65)	1.35 (0.83)	0.19	***0.014***
**BCVA at Month 1 <= 20/10, N(%)**	7 / 12 (58.3%)	2 / 5 (40.0%)	9 / 17 (52.9%)	3 / 8 (37.5%)	12 / 13 (92.3%)	15 / 21 (71.4%)	0.24	***0.030***
**BCVA at Month 3 (LogMAR), Mean (SD)**	1.29 (0.82)	0.20 (0.14)	1.07 (0.86)	0.64 (0.70)	2.10 (0.36)	1.44 (0.92)	0.37	***0.016***
**BCVA at Month 6 (LogMAR), Mean (SD)**	0.53 (0.63)	0.70 (NA)	0.56 (0.58)	0.7 (0.65)	1.76 (0.78)	1.31 (0.89)	***0.039***	***0.027***
**BCVA at Month 12 (LogMAR), Mean (SD)**	0.43 (0.60)	2 (NA)	0.62 (0.79)	0.72 (0.90)	1.88 (0.78)	1.25 (1.01)	0.21	***0.043***
**BCVA at last visit (LogMAR), Mean (SD)**	0.82 (0.99)	0.52 (0.67)	0.74 (0.91)	0.69 (0.85)	1.77 (0.75)	1.36 (0.94)	***0.028***	***0.009***
**Baseline vitritis grade (+), Mean (SD)**	1.62 (1.46)	2 (0)	1.72 (1.24)	2.25 (0.71)	2.58 (1)	2.45 (0.89)	**0.042**	0.18
**Number of retinal quadrants affected by VRN, Mean (SD)**	1.93 (1.22)	2.60 (1.52)	2.10 (1.29)	1.44 (1.05)	2.58 (1.38)	2.12 (1.36)	0.95	0.22
**Time between VRN onset and treatment (days), Mean (SD)**	54.20 (73.48)	7.0 (4.9)	38.47 (63.31)	4.86 (4.85)	11.38 (16.42)	9.10 (13.71)	**0.0046**	**0.0032**
**Duration of intravenous antivirals (days), Mean (SD)**	22.64 (19.94)	22 (12.53)	22.50 (18.16)	17.38 (5.45)	19.92 (8.90)	18.90 (7.64)	0.65	0.89
**IV Aciclovir, N (%)**	4 / 12 (33.3%)	5 / 5 (100%)	9 / 17 (52.9%)	4 / 8 (50%)	9 / 13 (69.2%)	13 / 21 (61.9%)	0.58	**0.064**

**IV Ganciclovir, N (%)**	8 / 13 (61.5%)	0 / 5 (0%)	8 / 18 (44.4%)	0 / 8 (0%)	0 / 13 (0%)	0 / 21 (0%)	**0.0007**	**0.0005**
**IV Foscarnet, N (%)**	6 / 13 (46.2%)	3 / 5 (60%)	9 / 18 (50%)	5 / 8 (62.5%)	10 / 13 (76.9%)	15 / 21 (71.4%)	0.17	0.49
**Number of Ganciclovir IVIs, Mean (SD)**	9.20 (10.86)	8.20 (4.27)	8.95 (9.54)	4.62 (2.83)	4.85 (5.98)	4.76 (4.93)	0.0816	0.32
**Number of Foscarnet IVIs, Mean (SD)**	1.67 (4.5)	1.6 (3.58)	1.65 (4.2)	1.62 (4.6)	7.23 (8.91)	5.1 (7.92)	0.088	**0.069**
**Retinal detachment, N (%)**	3 / 15 (20%)	0 / 5 (0%)	3 / 20 (15%)	2 / 8 (25%)	6 / 13 (46.2%)	8 / 21 (38.1%)	0.095	0.27
**Time to retinal detachment (days), Mean (SD)**	119 (183.23)	-	119 (183.23)	60 (84.85)	101.67 (122.45)	91.25 (110.05)	0.92	0.81
**Baseline viral load (log10 IU), Mean (SD)**	6.22 (1.06)	6.77 (0.98)	6.36 (1.04)	5.32 (1.54)	7.63 (1)	6.75 (1.66)	0.28	**0.0018**
**Evaluation time for slow or rapid responder status definition (weeks), Mean (SD)**	2.83 (3.27)	1.89 (0.27)	2.59 (2.84)	2.20 (0.30)	2.01 (0.32)	2.09 (0.32)	0.088	0.23
**Viral load kinetics at week 2 (rapid responder: >= 50% decrease in VL), N (%)**	7 / 13 (53.8%)	3 / 5 (60%)	10 / 18 (55.6%)	4 / 6 (66.7%)	5 / 12 (41.7%)	9 / 18 (50%)	0.74	0.82

HSV: herpes simplex virus; VZV: varicella zoster virus; CMV: cytomegalovirus; VNR: viral necrotizing retinitis; N: number; %: percentage; SD: standard deviation; IQR: 1st-3rd interquartile range; BCVA: best corrected visual acuity; IV: intravenous; IVI: intravitreal injection; Rapid responder: 50% or greater decrease of the baseline viral load after 2 +/-1 weeks of intravenous antiviral treatment. Vitritis grade was described according to the Nussenblatt classification [4].
[a] p-value of all (CMV+VZV) immunodepressed (ID) versus all (HSV2+VZV) immunocompetent (IC) patients or eyes, [b] p-value of CMV (ID) versus VZV (ID) versus HSV2 (IC) versus VZV (IC) patients or eyes.

Supplemental Table S3. Characteristics of patients and eyes in the subgroup of immunodepressed patients according to the cause of immune suppression

	**Immunodepressed patients (N=17) or eyes (N=20)**				
	**CMV**		**VZV**		**p-value [a]**
	**HIV**	**Other cause**	**HIV**	**Other cause**	
**Variables (described in terms of patients)**	**(N=3)**	**(N=10)**	**(N=1)**	**(N=3)**	
**Gender (females), N (%)**	1 / 3 (33.3%)	2 /10 (20%)	1 / 1 (100%)	1 / 3 (33.3%)	0.62
**Age (years), Mean (SD)**	40 (8.7)	65.5 (15.6)	44 (NA)	56.7 (12.1)	0.11
**Follow-up (months), Mean (SD)**	5.3 (5.1)	12.4 (11.9)	1 (NA)	6.7 (6.3)	0.39
**Bilateral retinal necrosis, N (%)**	2 / 3 (66.7%)	3 / 10 (30%)	1 / 1 (100%)	0 / 3 (0%)	0.15

**Time to bilateralization (months), Mean (SD)**	27.5 (28.9)	0 (0)	0 (NA)	-	-
**Variables (described in terms of eyes)**	**(N=3)**	**(N=12)**	**(N=2)**	**(N=3)**	
**Baseline BCVA (LogMAR), Mean (SD)**	0.4 (0.5)	1.0 (0.9)	0.9 (1.1)	0.9 (0.7)	0.79
**BCVA at Month 1 (LogMAR), Mean (SD)**	1.2 (1.1)	1.1 (0.9)	0.2 (0.2)	1 (0.9)	0.80
**BCVA at Month 1 <= 20/100, N (%)**	2 (66.7%)	5 (55.6%)	0 (0%)	2 (66.7%)	0.66
**BCVA at Month 3 (LogMAR), Mean (SD)**	1.8 (0.2)	1.1 (0.9)	-	0.2 (0.1)	0.22
**BCVA at Month 6 (LogMAR), Mean (SD)**	0.7 (NA)	0.5 (0.7)	-	0.7 (NA)	0.50
**BCVA at Month 12 (LogMAR), Mean (SD)**	0.6 (NA)	0.4 (0.6)	-	2 (NA)	0.21
**BCVA at last visit (LogMAR), Mean (SD)**	0.9 (1.2)	0.8 (0.9)	0.2 (0.2)	0.7 (0.9)	0.97
**Baseline vitritis grade (+), Mean (SD)**	0.5 (0.5)	1.9 (1.5)	2 (0)	2 (0)	0.24
**Number of retinal quadrants affected by VRN, Mean (SD)**	2.3 (1.5)	1.8 (1.2)	4 (0)	1.7 (1.1)	0.19
**Time between VRN onset and treatment (days), Mean (SD)**	67 (74.9)	51 (77.9)	3 (0)	9.7 (4.6)	0.07
**Duration of intravenous antivirals (days), Mean (SD)**	43.5 (51.6)	18 (5.5)	-	22 (12.5)	0.89
**IV Aciclovir, N (%)**	0 / 2 (0%)	4 / 10 (40%)	2 / 2 (100%)	3 / 3 (100%)	***0.054***
**IV Ganciclovir, N (%)**	3 / 3 (100%)	5 / 10 (50%)	0 / 2 (0%)	0 / 3 (0%)	***0.042***
**IV Foscarnet, N (%)**	1 / 3 (33.3%)	5 / 10 (50%)	0 / 2 (0%)	3 / 3 (100%)	0.28
**Number of Ganciclovir IVIs, Mean (SD)**	3 (3)	10.7 (11.6)	10 (0)	7 (5.6)	0.44
**Number of Foscarnet IVIs, Mean (SD)**	5 (8.7)	0.8 (2.9)	0 (0)	2.7 (4.6)	0.52
**Retinal detachment, N (%)**	0 / 3 (0%)	3 / 12 (25%)	0 / 2 (0%)	0 / 3 (0%)	1.0
**Time to retinal detachment (days), Mean (SD)**	-	119 (183.23)	-	-	
**Baseline viral load (log10 IU), Mean (SD)**	5.1 (1.4)	6.5 (0.8)	7.0 (0.1)	6.6 (1.3)	0.22
**Evaluation time for slow or rapid responder status (weeks), Mean (SD)**	1.3 (0.6)	3.2 (3.6)	1.8 (0.1)	1.9 (0.4)	0.40
**Viral load kinetics at week 2 (rapid responder: >= 50% decrease in VL), N(%)**	0 / 3 (0%)	7 / 10 (70%)	2 / 2 (100%)	1 / 3 (33.3%)	0.09

CMV: cytomegalovirus; VZV: varicella zoster virus; HIV: human immunodeficiency virus; VNR: viral necrotizing retinitis; N: number; %: percentage; SD: standard deviation; IQR: 1st-3rd interquartile range; BCVA: best corrected visual acuity; IV: intravenous; IVI: intravitreal injection; NA: not available; Rapid responder: 50% or greater decrease of the baseline viral load after 2 +/-1 weeks of intravenous antiviral treatment. Vitritis grade was described according to the Nussenblatt classification [4]. [a] p-value of CMV/HIV versus CMV/other cause versus VZV/HIV versus VZV/other cause of immunodepression.

Supplemental Table S4. Visual acuity evolution according to the causative virus

Variables (described in terms of eyes)	Virus				
**Variable**	**CMV (N=15)**	**HSV2 (N=8)**	**VZV (N=18)**	**Total (N=41)**	**p-value [a]**
**BCVA at baseline (LogMAR), Mean (SD)**	0.9 (0.8)	0.8 (0.7)	1.1 (0.8)	0.9 (0.8)	0.37
**BCVA at M1 (LogMAR), Mean (SD)**	1.1 (0.9)	0.6 (0.6)	1.4 (0.8)	1.2 (0.8)	0.06
**BCVA at M3 (LogMAR), Mean (SD)**	1.3 (0.8)	0.6 (0.7)	1.6 (0.9)	1.3 (0.9)	0.11
**BCVA at M6 (LogMAR), Mean (SD)**	0.5 (0.6)	0.7 (0.6)	1.6 (0.8)	1.0 (0.9)	
**BCVA at M12 (LogMAR), Mean (SD)**	0.4 (0.6)	0.7 (0.9)	1.9 (0.7)	0.9 (0.9)	
**Variation of BCVA between M1 and baseline (LogMAR), Mean (SD)**	0.04 (0.8)	-0.1 (0.5)	0.4 (0.9)	0.1 (0.8)	0.45
**Variation of BCVA between M3 and baseline (LogMAR), Mean (SD)**	0.2 (0.8)	-0.02 (0.7)	0.5 (1.1)	0.2 (0.9)	0.65
**BCVA≤ 20/100 at M1 (N,%)**	7 (58.3%)	3 (37.5%)	14 (77.8%)	24 (63.2%)	0.14
**BCVA≤ 20/100 at M3 (N, %)**	6 (75%)	2 (40%)	6 (75%)	14 (66.7%)	0.41

BCVA: best corrected visual acuity; HSV: herpes simplex virus; VZV: varicella zoster virus; CMV: cytomegalovirus; M: month; SD: standard deviation; N: number; %: percentage, [a] p-value of CMV versus HSV2 versus VZV.

Supplemental Table S5. BCVA evolution according to the occurrence of retinal detachment during the follow-up				
	Retinal detachment during follow-up			p-value
**Variables (described in terms of eyes)**	No (N =30)	Yes (N =11)	Total (N =41)	
**Baseline BVCA (LogMAR), Mean (SD)**	0.75 (0.70)	1.59 (0.74)	0.98 (0.80)	**0.0037** (Wilcoxon rank sum test)
**BVCA at month 1 (LogMAR), Mean (SD)**	0.99 (0.83)	1.74 (0.78)	1.19 (0.87)	**0.016** (Wilcoxon rank sum test)
**BCVA at month 1 ≤20/100, N(%)**	15 (53.6%)	9 (90%)	24 (63.2%)	**0.06** (Fisher's Exact Test for Count Data)
**BVCA at month 3 (LogMAR), Mean (SD)**	1.03 (0.91)	1.83 (0.56)	1.26 (0.89)	0.1253 (Wilcoxon rank sum test)
**BVCA at month 6 (LogMAR), Mean (SD)**	0.88 (0.80)	1.41 (0.92)	1.06 (0.86)	0.1159 (Wilcoxon rank sum test)
**BVCA at month 12 (LogMAR), Mean (SD)**	0.63 (0.77)	1.75 (0.90)	0.98 (0.95)	**0.024** (Wilcoxon rank sum test)
BCVA: best corrected visual acuity; SD: standard deviation; IQR: 1st-3rd interquartile range; N: Number.				

Supplemental Table S6. Logistic regression analysis of the predictive factors of retinal detachment			
**Univariate analysis**			
**Variable**	**OR**	**CI 95%**	**p-value**
**Age (years)**	1.22	0.98-1.51	0.058
**Immunodepression (yes)**	0.26	0.06-1.21	0.073
**BCVA at baseline (LogMAR)**	1.16	1.04-1.3	0.002
**Vitritis (+)**	2.26	1.01-5.05	0.028
**Number of IVI Ganciclovir > 5**	0.17	0.03-0.92	0.023
BCVA: best corrected visual acuity; OR: odds ratio; CI: confidence interval; IVI: intravitreal injection			
